# Osteopathy

**Published:** 1910-09-24

**Authors:** H. A. Barker


### EDITOR'S LETTER-BOX.

### OSTEOPATHY.

*To the Editor of* THE HOSPITAL.

SIR,—Some thirty-five years ago Hutton came to London and practised with extraordinary success the art of "bone-setting" in the West End. Of course, the doctors were unfriendly. They declared that he was a quack, just as they declared that Harvey was a mere *circulator*, and asserted that all Hutton's science lay in chance cures. But the movement thus instituted went on. There are now eight schools of bone-setting under Governmental recognition in the United States, with twelve thousand students who pay annually £40,000 in tuition fees alone. These startling facts have induced Dr. Alexander Bryce, of Glasgow and Cambridge, to take the matter into study. He admits that he has been interested in the bone-setter's methods of treatment because of the "remarkable improvement" in some of his "own reputedly incurable patients." And after careful examination he has come to the conclusion that there must be "virtue in a method which has such vitality as to spread all over a continent in a few years, and, at the present rate of progress, bids fair to travel all over the world." Dr. Bryce points to the fact that Dr. Wharton Hood, after a thorough investigation of the methods of the late Mr. Robert Hutton, declared that his experiences were invaluable and the results astounding. But Dr. Bryce, while fighting for "the admission of this new form of scientific bone-setting among the recognised methods of treatment practised by the medical profession," is not as generous to me as was Dr. Wharton Hood to Mr. Hutton.

Yet for nearly twenty years, as Hutton's successor, and after operating on over thirty thousand cases, I have repeatedly exhorted the faculty to investigate this important branch of therapeutics. Almost four years ago I wrote to the medical papers offering to submit these methods to any test before an authorised committee of qualified surgeons, but no heed was given to my plea.

I beg of your, Sir, to insert this letter in your columns, and thus direct the attention of surgeons to Dr. Bryce's paper on the subject which is of such vital importance to suffering humanity, and which the *Lancet* years ago described as a "neglected corner of the domain of surgery."

Yours faithfully,

H. A. BARKER.

